# Vessel wall MR imaging of aortic arch, cervical carotid and intracranial arteries in patients with embolic stroke of undetermined source: A narrative review

**DOI:** 10.3389/fneur.2022.968390

**Published:** 2022-07-28

**Authors:** Yu Sakai, Vance T. Lehman, Laura B. Eisenmenger, Emmanuel C. Obusez, G. Abbas Kharal, Jiayu Xiao, Grace J. Wang, Zhaoyang Fan, Brett L. Cucchiara, Jae W. Song

**Affiliations:** ^1^Department of Radiology, Hospital of the University of Pennsylvania, Philadelphia, PA, United States; ^2^Department of Radiology, The Mayo Clinic, Rochester, MN, United States; ^3^Department of Radiology, University of Wisconsin-Madison, Madison, WI, United States; ^4^Department of Radiology, Cleveland Clinic, Cleveland, OH, United States; ^5^Department of Neurology, Cerebrovascular Center, Neurological Institute, Cleveland, OH, United States; ^6^Department of Radiology, Keck School of Medicine, University of Southern California, Los Angeles, CA, United States; ^7^Department of Vascular Surgery and Endovascular Therapy, Hospital of the University of Pennsylvania, Philadelphia, PA, United States; ^8^Department of Neurology, Hospital of the University of Pennsylvania, Philadelphia, PA, United States

**Keywords:** vessel wall MRI, atherosclerosis, imaging, cerebrovascular disease/stroke, embolic stroke of undetermined source (ESUS), stroke

## Abstract

Despite advancements in multi-modal imaging techniques, a substantial portion of ischemic stroke patients today remain without a diagnosed etiology after conventional workup. Based on existing diagnostic criteria, these ischemic stroke patients are subcategorized into having cryptogenic stroke (CS) or embolic stroke of undetermined source (ESUS). There is growing evidence that in these patients, non-cardiogenic embolic sources, in particular non-stenosing atherosclerotic plaque, may have significant contributory roles in their ischemic strokes. Recent advancements in vessel wall MRI (VW-MRI) have enabled imaging of vessel walls beyond the degree of luminal stenosis, and allows further characterization of atherosclerotic plaque components. Using this imaging technique, we are able to identify potential imaging biomarkers of vulnerable atherosclerotic plaques such as intraplaque hemorrhage, lipid rich necrotic core, and thin or ruptured fibrous caps. This review focuses on the existing evidence on the advantages of utilizing VW-MRI in ischemic stroke patients to identify culprit plaques in key anatomical areas, namely the cervical carotid arteries, intracranial arteries, and the aortic arch. For each anatomical area, the literature on potential imaging biomarkers of vulnerable plaques on VW-MRI as well as the VW-MRI literature in ESUS and CS patients are reviewed. Future directions on further elucidating ESUS and CS by the use of VW-MRI as well as exciting emerging techniques are reviewed.

## Introduction

The term Embolic Stroke of Undetermined Source (ESUS) was introduced in 2014 by the Cryptogenic Stroke/ESUS International Working Group (1). It describes patients with non-lacunar brain infarcts without a high-grade large artery stenosis, a high-risk cardioembolic source, or another determined stroke mechanism. The ESUS population is of clinical importance as it may account for up to 20% of all ischemic strokes (1).

**Figure 1:**
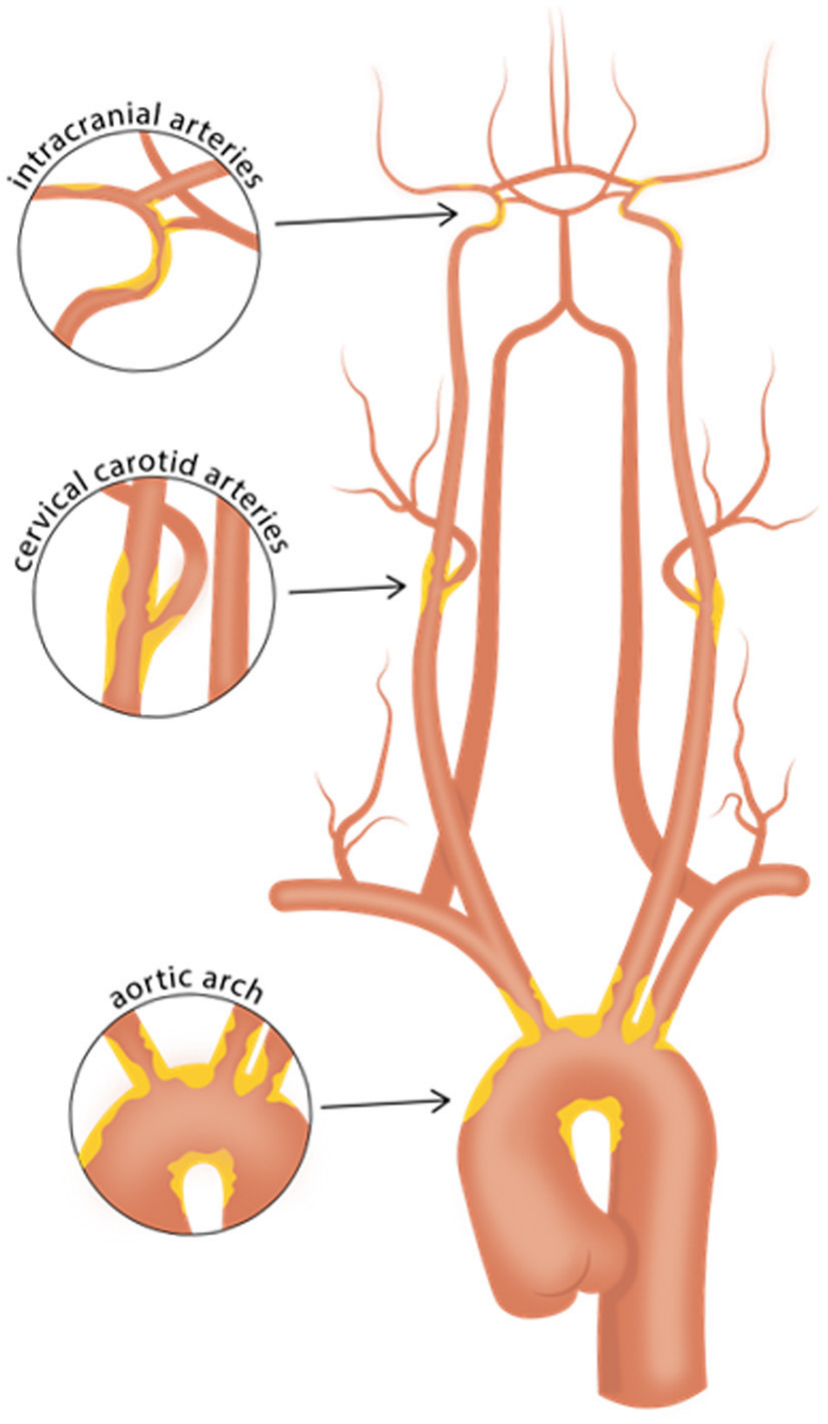
Vascular beds commonly affected with atherosclerosis. Careful evaluation of high-risk plaque features using vessel wall MRI of the intracranial arteries, cervical carotid arteries, and aortic arch may aid in identifying culprit plaques in patients with cryptogenic stroke or embolic stroke of undetermined source. Such efforts may help identify causes of stroke that may have been previously overlooked.

As prior clinical trials with patients initially suspected of cryptogenic stroke (CS) revealed a high prevalence of underlying atrial fibrillation (2, 3), subsequent clinical trials focusing on the ESUS population, namely NAVIGATE ESUS and RE-SPECT ESUS, studied the use of anticoagulant therapy, with the underlying premise that unrecognized paroxysmal atrial fibrillation might be a common ESUS mechanism. However, both of these randomized clinical trials showed no significant reduction in recurrent stroke with anticoagulation compared to antiplatelet therapy (4, 5). Given this result, the prospect that non-cardiogenic embolic sources contribute to a significant proportion of ESUS seems increasingly likely. One such source is non-stenosing plaque, which might be present in the cervical carotid or vertebral arteries (6, 7), the intracranial arteries (8), or the aortic arch (9) (Figure 1). In the carotid arteries, this concept has been recently referred to as “symptomatic non-stenotic carotid disease” (SyNC) (10).

Recent advancements in neuroimaging allow us to better evaluate non-stenotic plaque and characterize its potential embolic risk. Potential plaque imaging biomarkers include wall thickness, intraplaque hemorrhage (IPH), lipid-rich necrotic core (LRNC), fibrous cap status, neovascularization manifested by enhancement, and surface morphologies such as ulcerations (11–13). In particular, vessel wall MRI (VW-MRI) is increasingly being used for evaluation of plaque to identify vulnerable features. VW-MRI protocols commonly include magnetic resonance angiography (MRA) and multi-contrast MRI sequences that suppress the signal from adjacent tissue and flowing blood to highlight vessel wall pathologies of both intracranial and extracranial vessels. VW-MRI complements traditional imaging techniques such as CT angiography, MRA, and Digital Subtraction Angiography (DSA) because it allows evaluation of pathology beyond luminal stenosis, namely vulnerable plaque features such as IPH, LRNC, and fibrous cap status (13).

We review how VW-MRI has been explored for evaluation of disease in the cervical carotid arteries, intracranial arteries, and the aortic arch in ischemic stroke patients and show imaging examples of vulnerable plaque features. We also review the available literature on how VW-MRI has been used to study patients who meet criteria for ESUS (or, in older literature prior to introduction of the ESUS concept, CS).

### Search strategy

We searched PubMed for reports published between January 1980 to March 1st, 2022. Search terms included: “Vessel Wall Imaging,” “Vessel Wall MR Imaging,” “High Resolution Vessel Wall MRI,” “Stroke Etiology,” “Cryptogenic Stroke,” “Embolic source of undetermined source,” “ESUS,” “Atherosclerosis,” “Atherosclerotic Plaque,” “Intraplaque hemorrhage,” and “Lipid-rich necrotic core.” Furthermore, we reviewed the reference lists of retrieved reports to identify additional relevant articles. Relevant practice guidelines and their reference lists were also reviewed. We did not restrict our search by language. The final reference list was generated based on relevance to the broad scope of this narrative review with preference given to meta-analyses/systematic reviews and studies with rigorous methodology.

## Cervical carotid arteries

### Background

Established in 1993, the TOAST classification remains the most widely used system of classifying stroke by etiology. The TOAST classification applies a threshold of ≥50% carotid luminal stenosis as causal of stroke and classifies patients with <50% stenosis and no other identified mechanism as having “stroke of undetermined cause” (14). This is problematic as it may result in an underestimation of the role of carotid atherosclerosis in ischemic stroke since patients with non-stenosing but potentially culprit plaques are not captured. A more recent classification system, the ASCOD Phenotyping of Ischemic Stroke, has expanded the definition of carotid-etiology by classifying the presence of an ipsilateral atherosclerotic stenosis <50% in an intra- or extracranial artery with a luminal thrombus supplying the ischemic field as a potential stroke etiology (15).

The concept of “vulnerable plaque” was initially established with regards to the coronary arteries, where several features of coronary plaques were found to be associated with acute coronary events and sudden cardiac death. These high-risk features include a large lipid rich necrotic core (LRNC), thin/ruptured fibrous cap, and intraplaque hemorrhage (IPH) (16–18).

Advancements in imaging modalities have allowed us to extend the concept of the vulnerable plaque to the evaluation of the carotid arteries beyond assessing the degree of stenosis to explore imaging features and potential biomarkers such as IPH, LRNC, and fibrous cap status (19–21). In particular, MRI has been validated with histology to have high sensitivity and specificity in the evaluation of these vulnerable plaque features (22–24). In recent years, increasing interest in the use of VW-MRI to assess the carotid arteries in ESUS/CS patients has led to investigations on potential imaging biomarkers that may aid us in deciphering ESUS/CS and improve risk stratification. Below we review several plaque features that have been investigated as promising biomarkers.

### Carotid plaque features

Studies to date using VW-MRI to evaluate carotid plaque features in the ESUS population have examined one or more of the following imaging biomarkers: IPH, fibrous cap rupture, LRNC, and thrombus. We briefly review how MRI identifies these specific plaque characteristics.

#### Intraplaque hemorrhage

**Figure 2:**
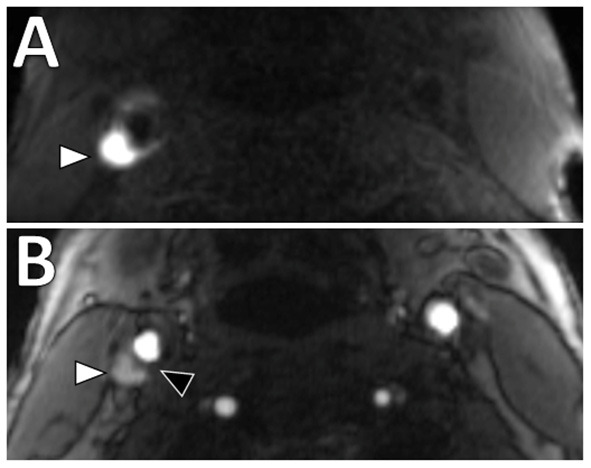
Intraplaque hemorrhage in carotid plaque on Magnetization-Prepared Rapid Gradient Echo (MPRAGE) and Time-of-flight MR angiography (TOF MRA). T1 hyperintense IPH at the right carotid bifurcation (white arrowheads) on both **(A)** MPRAGE and **(B)** TOF MRA. The MPRAGE image has fat and blood flow suppression, allowing for the IPH to standout in contrast to the vessel lumen and surrounding soft tissues. The TOF MRA image also demonstrates an intact hypointense fibrous cap at this level (black arrowhead).

Intraplaque hemorrhage (IPH) is one of the features of a vulnerable plaque (18). Physiologically, it represents the extravasation of red blood cells or iron accumulation in plaque, which may result in plaque instability (25). IPH in carotid plaque has been shown to have significant association with ischemic stroke (26, 27). A meta-analysis on carotid plaque MRI and stroke risk reported patients with carotid IPH on MRI were almost five-times more likely to have subsequent stroke or transient ischemic attack than those without (hazard ratio 4.59, 95% confidence interval 2.91–7.94), although a limitation of the included studies was a considerable range in stenosis degree (28). On imaging, T1-weighted (T1W) sequences are considered the best imaging modality for detection of IPH in the carotid arteries compared to CT or ultrasound due to its ability to detect blood products (11, 13). A variety of T1W sequences can be used, including magnetization-prepared rapid acquisition gradient echo (MPRAGE) and 3D time-of-flight (TOF) sequences (29, 30). An example is shown in Figure 2. MPRAGE is considered to have the highest accuracy given other plaque components such as fibrous tissue and LRNC are suppressed on the inversion-recovery preparation and fat saturation of MPRAGE sequences thereby allowing for the highest tissue contrast between IPH and background structures. Using histology as the gold standard, MPRAGE has the highest specificity (97%) and sensitivity (80%) in detecting carotid IPH (31).

#### Lipid rich necrotic core

Physiologically, LRNC in carotid plaques represent heterogeneous tissue composed of apoptotic cell debris, calcium particles, and cholesterol crystals (19). A recent meta-analysis showed carotid plaque LRNC is associated with increased risk of ipsilateral ischemic stroke (28). On MRI, LRNC can be seen as focal hypointensity on a T2-weighted sequence. Contrast-enhanced T1-weighted sequences can also help distinguish non-enhancing LRNC from enhancing fibrous plaque tissue (22). LRNC and IPH commonly co-exist and can appear similarly on CT as low-density plaque. While there are efforts to further differentiate the two by Hounsfield units (32, 33), MRI is generally considered superior (19).

#### Fibrous cap status

Physiologically, the fibrous cap refers to a layer of fibrous connective tissue that contains macrophages and smooth muscle cells, which if ruptured, exposes the adjacent LRNC to luminal blood resulting in activation of the thromboembolic cascade. It has been shown that thin or ruptured fibrous cap is associated with increased risk of ischemic stroke (27, 28, 34). One of the key advantages of MRI is that it can assess fibrous cap status with contrast-enhanced T1W sequences and 3D time-of-flight magnetic resonance angiography (TOF MRA) (11). On contrast-enhanced T1W sequences, normal thick fibrous cap shows smooth linear enhancement overlying the plaque, whereas on TOF MRA, uniform hypointense band between bright lumen and gray plaque core is present (22, 24). An example is shown in Figure 2B. Presence of an irregular and disrupted fibrous cap is associated with ipsilateral ischemic stroke, most commonly due to a thromboembolic mechanism (35). However, this feature can be more difficult to assess than other features in clinical practice.

#### Surface morphology and ulceration

**Figure 3:**
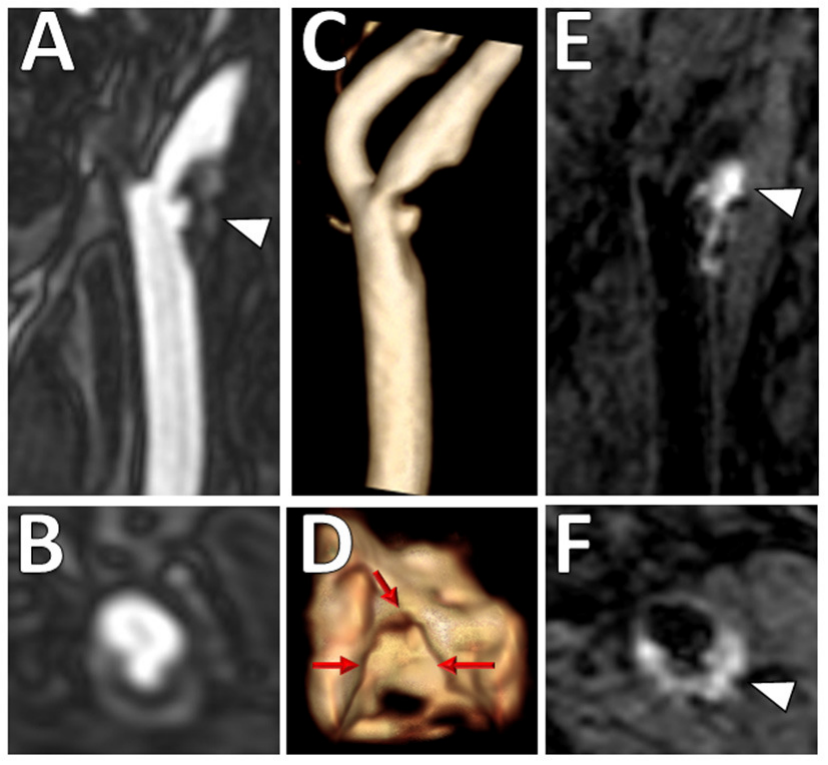
Ulcerated plaque with intraplaque hemorrhage on VW-MRI. Male patient presented with clinical and imaging evidence of a 1–2-year history of recurrent left MCA territory infarcts. **(A,B)** Contrast-enhanced MRA neck confirmed a left cervical carotid bifurcation stenosis with an ulcerated plaque (**A**, arrowhead). **(C,D)** 3D surface volume rendering of the left carotid plaque depicts the ulcerated surface morphology with **(D)** axial image through the ulceration showing marked surface irregularity (red arrows). **(E,F)** Carotid VW-MRI showed intraplaque hemorrhage (arrowheads), depicted as T1 hyperintense signal on a fat-suppressed T1W MPRAGE image. Based on the clinical and imaging findings, the patient underwent a left carotid endarterectomy.

Luminal surface morphology of carotid plaques can be classified as smooth (no irregularity/ulceration), irregular (surface fluctuates from 0.3 to 0.9 mm), or ulcerated (cavities measuring ≥1 mm) (36). Both irregular and ulcerated carotid plaque surfaces are associated with increased risk of stroke (36, 37). An example is shown in Figure 3. On MRI, luminal evaluation is possible with contrast-enhanced MRA (38, 39). MRI can detect carotid plaque ulcerations with similar sensitivity to CTA and the use of contrast-enhanced MRA is preferred over unenhanced TOF MRA due to reduced flow artifacts (38).

### Literature on carotid VW-MRI in ESUS patients

Vessel wall-MRI of carotid plaques in the ESUS population suggests a higher prevalence of vulnerable plaque features in the ipsilateral carotid artery compared to the contralateral, regardless of the degree of luminal narrowing (35, 40–45) (Table 1). These observations support the hypothesis that embolization from large artery atherosclerosis may occur even in the absence of hemodynamically significant internal carotid artery stenosis.

**Table 1:** Select studies using VW-MRI in an ESUS/CS population for evaluation of carotid atherosclerosis.

**Study**	**Imaging technique**	**Vessel**	**Key imaging characteristics**	**Select results**
Altaf et al. (26)	1.5 T. 3D T1W GRE	Carotid	IPH	39 (61%) ipsilateral arteries with IPH. During follow-up, 13 ischemic events, of which 5 were strokes, occurred in those with ipsilateral carotid IPH (HR = 9.8, 95% CI 1.3–75.1, *p* = 0.03)
Freilinger et al. (35)	3T. TOF MRA, axial pre-and post-contrast black-blood T1W, PD, T2W	Carotid	IPH, fibrous plaque rupture, luminal thrombus	AHA-LT V1 plaques in 37.5% ipsilateral to the stroke, none in contralateral (*p* = 0.001). Most common diagnostic feature of AHA-LT V1 plaques: IPH (75%), fibrous plaque rupture (50%), luminal thrombus (33%)
Bayer-Karpinska et al. (40)	3T. TOF MRA, axial pre- and post-contrast black-blood T1W, PD, T2W. Additional Dynamic contrast-enhanced MRI series in subgroup	Carotid	Characteristics including fibrous cap rupture, IPH, juxtaluminal hemorrhage/mural thrombus	Initial analysis showed significantly higher prevalence of complicated ipsilateral AHA-LT VI plaques in cryptogenic stroke patients than contralateral (37% vs. 3%, *p* < 0.0001)
Gupta et al. (41)	1.5T/3T. 3D TOF MRA	Carotid	IPH	6 patients (22.2%) with IPH ipsilateral to the side of ischemic stroke, 0 patients with IPH on contralateral side (*p* = 0.01)
Gupta et al. (42)	1.5T/3T. 3D TOF MRA	Carotid	IPH	22 patients (20.2%) with <50% ICA plaque with IPH ipsilateral to stroke, 9 (8.3%) patients with IPH in <50% ICA plaque contralateral to side of stroke (*p* = 0.01)
Hyafil et al. (43)	3T. 3D TOF MRA, axial pre- and post-contrast black-blood T1W, T2W	Carotid	IPH, fibrous cap rupture, luminal thrombus	AHA-LT V1 plaques significantly higher ipsilateral to the stroke side than contralateral (39 vs. 0 %; *p* = 0.001). For all other AHA lesion types, no significant differences between ipsilateral and contralateral sides
Singh et al. (44)	3T. 3D T1W GRE	Carotid	IPH	Significantly higher prevalence of ipsilateral than contralateral IPH (20% vs. 8.6%, *p* = 0.005)

*VW-MRI, vessel wall MRI; T1W GRE, T1 weighted gradient-echo; T, Tesla; TOF MRA, Time-of-flight MR angiography; PD, proton density; T2W, T2 weighted; IPH, intraplaque hemorrhage; AHA-LT VI, American heart association lesion type VI; HR, hazard ratio; 95% CI, 95% confidence interval*.

Two recent systematic reviews/meta-analyses described the prevalence of carotid artery IPH and other high-risk features in mildly stenotic carotid arteries on VW-MRI in patients meeting ESUS criteria (6, 7). Mark et al., included seven studies from 2012 to 2020 with 354 patients and reported a prevalence estimate of IPH ipsilateral to cerebral ischemia to be 25.8% (95% CI 13.1–38.5) and odds ratio of IPH ipsilateral to ischemia vs. contralateral side to be 6.92 (95% CI 3.04–15.79). Pooled analyses by Kamtchum-Tatuene et al., included eight studies from 2013 to 2018 with 323 patients with unilateral anterior circulation ischemic stroke with plaque imaging performed within 14 days of stroke onset using either VW-MRI, CTA, or US (7). High-risk features assessed included ulceration, IPH, thrombus, fibrous cap rupture, echolucency, or plaque thickness ≥3 mm. They reported the prevalence of mild (≤50% luminal narrowing) carotid stenosis with the aforementioned high-risk features in the ipsilateral carotid to be 32.5% (95% CI, 25.3–40.2) compared to 4.6% (95% CI 0.1–13.1) in the contralateral side. Plaque with high-risk features was more than five times more likely to be present in the ipsilateral vs. contralateral carotid artery (OR 5.5, 95% CI, 2.5–12.0). Both studies highlight a role for VW-MRI to detect the presence of morphologic and compositional features in mildly stenotic plaque in patients with ESUS/CS.

### Future directions

The use of VW-MRI to evaluate carotid plaque features is a rapidly evolving field. Multi-center prospective studies with larger patient pools to clarify the relationship between vulnerable carotid plaque features on VW-MRI and risk of stroke recurrence are needed. Currently, there are several prospective studies (CAPIAS, CARE II, and the PARISK) intended to examine the value of carotid plaque imaging. These studies are described in more detail in Table 2 (40, 46, 47). How to use diagnostic imaging information from carotid VW-MRI in clinical decision-making to determine stroke mechanism or to choose optimum preventative therapy remains an area of investigation. Some challenges to clinical translation of these VW-MRI protocols include lengthy acquisition times for multiple contrast weightings. Technical efforts to accelerate or simplify protocols include testing different neurovascular vs. surface coils (48) and the use of simultaneous non-contrast angiography and intraplaque hemorrhage (SNAP) (49) and multi-contrast atherosclerosis characterization (MATCH) (50) techniques and remain an active area of investigation.

**Table 2:** Prospective carotid plaque imaging studies.

**Study**	**Study sites, start dates**	**Patient selection and imaging**	**Primary outcome**	**Secondary outcome**
The carotid plaque imaging in acute stroke study (CAPIAS) (40)	• Initiated Feb 2011 • 3 Sites: Interdisciplinary Stroke Center in Munich (Ludwig-Maximilians-University), Technical University Munich, University of Freiburg • Observational cohort study; • NCT01284933	• Age >49 years • Stroke or TIA with symptom onset within 7 days • 1 or more acute ischemic lesion(s) on DWI in the territory of a single internal carotid artery • <70% stenosis by NASCET in carotid artery ipsilateral to stroke or TIA defined by US • Carotid artery plaques in the ipsi- or contra-lateral carotid artery as defined by ultrasound (plaque thickness at least 2 mm; located within 1 cm proximal or distal to the carotid bifurcation) • Excluded if history of neck radiation, DWI positive lesions outside territory of a single ICA; surgery within 24 hours prior to MRI • All subjects imaged with VW-MRI at baseline and 12 months follow-up • Subgroup imaged with dynamic CE VW-MRI to visualize neovascularization and inflammation • Subgroup imaged with ^18^F-FDG PET/MRI at baseline to quantify plaque inflammation	Prevalence of complicated AHA-LT VI plaques	• Association of AHA-LT VI plaques with recurrence rates of ischemic events up to 36 months • Rates of new ischemic lesions on cerebral MRI (including clinically silent lesions) after 12 months • Influence of specific AHA-LT VI plaque features on the progression of atherosclerotic disease burden, infarct patterns, biomarkers and aortic arch plaques
Chinese Atherosclerosis Risk Evaluation (CARE II) (46)	• Sites: 13 medical centers and hospitals in China and University of Washington • Cross-sectional study; • NCT02017756	• Stroke or TIA within 2 weeks • Carotid plaque in at least 1 carotid artery with wall thickness ≥1.5, as defined by US • Exclude cardiogenic stroke, hemorrhagic stroke, neck radiation, unable to undergo MRI • Carotid VW-MRI and routine brain MRI	Prevalence and characteristics of specific VW-MRI features of high-risk atherosclerotic plaque in Chinese patients with stroke or TIA	• Association of carotid plaque features and cerebral infarcts • Differences of carotid plaque patterns among different regions in China • Gender specific characteristics of carotid plaque in Chinese patients with stroke
Plaque At RISK (PARISK) (47)	• Observational cohort study • 4 Sites: Academic Medical Center Amsterdam; Erasmus Medical Center Rotterdam; Maastricht University Medical Center; University Medical Center Utrecht	• TIA, amaurosis fugax or minor stroke (modified Rankin scale ≤3) of the carotid artery territory and an atherosclerotic plaque with <70% stenosis of the ipsilateral ICA • No revascularization procedure • Exclude cardioembolic course, clotting disorder, unable to undergo MRI with contrast • Imaging performed within 5-day window of symptom onset • Baseline: Carotid VW-MRI, MRI brain, CTA, TCD, US, & biomarkers • 2 years: Carotid VW-MRI (subset), CTA, TCD, Carotid US, Brain MRI (all)	Identify whether VW-MRI, multidetector CTA, US and/or transcranial Doppler will predict future ischemic events in symptomatic patients with <70% carotid stenosis Endpoint: ipsilateral recurrent ischemic stroke or TIA and/or ipsilateral ischemic brain lesion on follow-up brain MRI	• Identify determinants for plaque progression • Examine relationship between plaque characteristics, microemboli, and vascular damage on brain MRI • Determine associations between blood biomarkers and plaque parameters

*VW-MRI, vessel wall MRI; TIA, transient ischemic attack; US, ultrasound, MRI, magnetic resonance imaging, AHA-LT VI, American Heart Association-Lesion Type VI; CTA, computed tomography angiography; TCD, transcranial doppler; US, ultrasound*.

## Intracranial arteries

### Background

Several structural differences between intracranial and extracranial arteries may contribute to differences in plaque composition and the development of vulnerable plaque features. Intracranial arteries have denser internal elastic lamina, a thinner media, less abundant adventitia with decreased elastic fibers and do not have external elastic lamina (51). In addition, there is a relative paucity of vasa vasorum in the walls of intracranial arteries (52), possibly because intracranial arteries are bathed in nutrient-rich CSF (53). The development and progression of atherosclerotic lesions may therefore be different between intracranial and extracranial arteries (54–56). For example, fibrosis is more prevalent than lipid infiltration of the intima or the adventitia in intracranial plaque compared to extracranial plaque (57). Much of our understanding of imaging features of atherosclerosis stems from extracranial carotid arteries due to the availability of carotid endarterectomy specimens and histologic validation studies (58). Given the lack of readily available specimens for intracranial arteries, intracranial VW-MRI may be a non-invasive imaging modality enabling serial imaging to provide additional insight about intracranial atherosclerosis. Several features of intracranial atherosclerosis on VW-MRI have been described (59, 60) and are detailed below.

### VW-MRI of intracranial plaque features

#### Intraplaque hemorrhage

**Figure 4:**
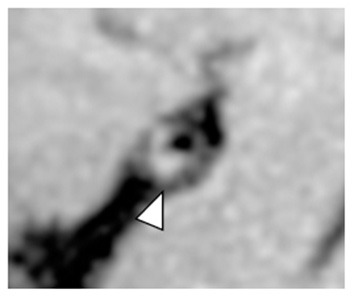
Intrinsic T1 hyperintense signal of intracranial plaque on precontrast VW-MRI. Orthogonal view of a precontrast VW-MRI of the middle cerebral artery shows intrinsic T1 hyperintense signal (arrowhead) and positive (outward) wall remodeling (arrowhead).

Presence of intrinsic T1 hyperintensity on T1W imaging of intracranial plaque is thought to reflect IPH (Figure 4). More specifically, IPH has been defined by several authors as having T1 signal intensity of >150% of the adjacent gray matter (61, 62) or adjacent muscle, noting wide variability of referenced tissue (59). In one case report, radiology and pathology findings were indeed histologically validated as such (63). Similar to the cervical carotid arteries, IPH may also be associated with ipsilateral ischemic stroke. A meta-analysis pooling five studies included 521 intracranial vessel wall segments of the circle of Willis and reported an odds ratio of 2.1 (95% CI 1.3, 3.3) for the presence of T1W hyperintensity in a culprit plaque on VW-MRI in patients with acute to subacute ischemia in the territory supplied by that culprit plaque (64). Notably, many of the included studies originated in China and presumably predominantly included Chinese patients. In Chinese studies, the reported prevalence of IPH ranges between 12 and 30% (65, 66) and appears to be higher compared to the Western counterpart. These studies raise the possibility of differences in plaque composition by race for extracranial vs. intracranial plaque (67) and additional studies evaluating ethnic differences would be valuable.

#### LRNC

The prevalence of LRNC as part of intracranial atherosclerosis in ischemic stroke patients is unclear. Due to the restraints of spatial resolution, detecting LRNC on intracranial VW-MRI is a challenge. However, several radiology-pathology correlation studies have histologically validated the presence of LRNCs in intracranial plaques indicating a contributory role in plaque progression and a feature of vulnerability (68–71). In these studies, LRNC showed T1W hypointensity on fat-suppressed T1W VW-MRI, Short TI inversion recovery (STIR) hypointensity, T1 iso/hyperintensity and T2 iso/hypointensity.

#### Vessel wall thickening

Vessel wall thickening due to atherosclerotic plaque, has been described as a common feature of intracranial atherosclerosis on VW-MRI, albeit non-specific, as wall thickening is also seen in other intracranial pathologies such as vasculitis (12, 72).

**Figure 5:**
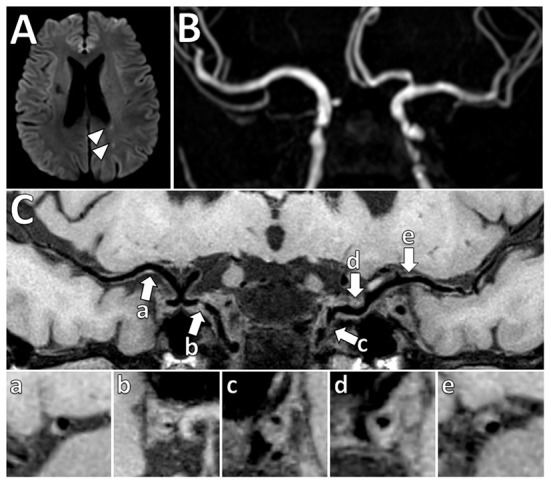
Vessel wall thickening and atherosclerosis on VW-MRI in a symptomatic patient. **(A)** A patient with left parietal lobe acute ischemic infarcts (arrowheads) underwent intracranial VW-MRI. **(B)** Time-of-flight MRA and **(C)** intracranial VW-MRI shows multiple intracranial plaques (arrows) that showed both eccentric (a, b, c, e) and concentric (d) vessel wall thickening. The culprit lesion was thought to be the most stenotic lesion (c) in the left internal carotid artery.

The pattern of wall thickening can be either concentric or eccentric. Per a recent systematic review, the most common definition of identifying intracranial plaque using VW-MRI was focal/eccentric vessel wall thickening (59). Several authors have defined that vessel wall thickening can be considered concentric if it is circumferential and uniform, with the thinnest segment being at least 50% of the thickest segment. On the other hand, vessel wall thickening is considered eccentric if the wall thickening is clearly focal, or when circumferential wall thickening is noted but the thinnest segment of the wall thickening is <50% of the thickest segment (62, 73). Examples of plaque with both eccentric and concentric wall thickening are shown in Figure 5.

#### Contrast enhancement

**Figure 6:**
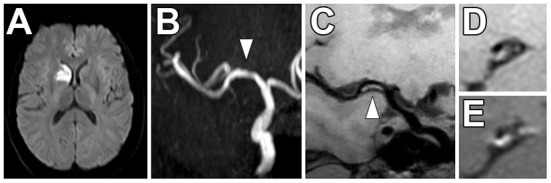
Non-stenotic right middle cerebral artery enhancing culprit plaque. **(A)** A patient with a right basal ganglia acute infarct showed **(B)** mild luminal irregularity but no appreciable stenosis of the right middle cerebral artery on time-of-flight MRA imaging (arrowhead). **(C)** Precontrast VW-MRI showed eccentric wall thickening along the right M1 middle cerebral artery (arrowhead). **(D)** Precontrast and **(E)** postcontrast images in the orthogonal plane through the plaque shows eccentric wall thickening and enhancement of the culprit plaque, which likely caused the ischemic infarct.

Multiple VW-MRI studies on intracranial atherosclerosis have shown that there is a higher prevalence of ipsilateral ischemic stroke in patients with contrast enhancing intracranial plaques independent of the degree of luminal stenosis (74–80). Figure 6 shows an example of an enhancing culprit plaque in a non-stenotic vessel that was felt to be the most likely source of a right basal ganglia ischemic stroke after a diagnostic work-up. VW-MRI studies with histological correlation propose contrast enhancement is a marker of inflammation and instability (70, 71). A meta-analysis pooling 990 circle of Willis vessel segments from 11 VW-MRI studies reported that it was more than seven times more likely to observe contrast enhancement of a culprit plaque in association with acute/subacute ischemia in the supplied vascular territory {OR of 7.4 [(95% CI, 3.4–16.4), *p* < 0.001]} (64), indicating this may be one of the stronger imaging biomarkers to detect culprit intracranial plaque. In the literature, definitions of focal/eccentric and circumferential vessel wall thickening detailed above are similar to the definitions of degrees of eccentric and circumferential enhancement.

#### Vessel wall remodeling

There are two features of vessel wall remodeling, outward and inward, which may be associated with different risks of ischemic events. Arterial walls can accommodate plaque deposition by adapting and remodeling outwardly (81), also known as positive wall remodeling (12). Initial studies in the coronary arteries suggested positive wall remodeling is associated with features of plaque instability (82). A meta-analysis pooling 352 middle cerebral and basilar artery segments from seven VW-MRI studies reported an odds ratio of 5.6 [(95% CI, 2.2–14.0), *p* < 0.001] for presence of positive wall remodeling in culprit plaques supplying the ischemic territory (64). In contrast, fibrotic healing changes may result in arterial wall shrinkage, otherwise known as negative remodeling (81, 83) and hypoperfusion due to stenosis may be a stroke etiology. Additional efforts to understand the role of intracranial vessel wall remodeling in ischemic events are warranted.

#### Calcifications

The role of calcification in intracranial atherosclerosis is unclear. Atherosclerotic calcification burden is thought to be a marker for cardiovascular events. CT is the gold standard for detecting intracranial calcifications. Detecting calcifications on MRI can be difficult as signal intensities may vary (84, 85), though most often are described to be hypointense on 3D TOF, T1W, T2W, proton density sequences and non-enhancing on gadolinium-enhanced T1W sequence (86). Although CT may be best for calcification detection, distinguishing the type of vascular calcification may be a challenge. For instance, calcifications affecting the arterial intima vs. media may have different clinical consequences. Intimal calcifications are associated with subintimal lipid and cholesterol deposition and macrophage accumulation whereas calcifications of the media are metabolite-induced vascular changes in the absence of lipid deposits and contribute to arterial stiffness (87, 88). Intracranial VW-MRI may play a role to help discriminate intimal calcifications. In a study with 75 patients, non-contrast CT and VW-MRI were used to evaluate intracranial artery calcification and intracranial atherosclerotic plaques, respectively. The study reported 72% of intimal calcifications coexisted with atherosclerotic plaques whereas only 10.2% of medial calcifications coexisted with atherosclerotic plaques (89). The authors also reported intimal calcifications were more common in non-culprit plaques (25.9 vs. 9.4% *P* = 0.008) in their study cohort, raising the possibility that intimal calcifications may indicate a stable form of plaque (89). Additional studies would be of value to understand intracranial calcifications with VW-MRI having a complementary role to CT.

### Intracranial VW-MRI in ESUS patients

**Figure 7:**
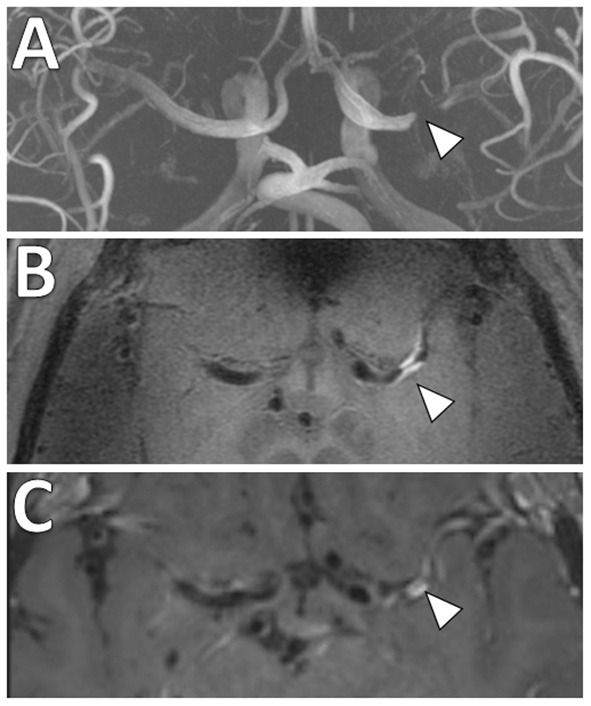
Comparison of 3 and 7 Tesla (T) VW-MRI for intracranial atherosclerosis. A patient with history of obesity, hyperlipidemia and hypertension and ischemic stroke underwent a **(A)** 7T TOF-MRA, which showed left M1 middle cerebral artery severe stenosis (arrowhead). **(B)** 7T MRI axial T1 SPACE post-gadolinium (0.5 mm isotropic resolution) image clearly delineates arterial wall thickening and avid vessel wall enhancement in the region of stenosis (arrowhead). **(C)** The same patient was imaged 18 days later on a 3T MRI (T1 SPACE post-gadolinium, 0.8 × 0.8 × 1.0 mm) to assess the stenosis (arrowhead). The 7T VW-MRI shows improved conspicuity and delineation of the margins of the arterial wall compared to the 3T, an advantage of the higher magnet strength due to the higher signal to noise leveraged for higher spatial resolution and soft tissue contrast.

There are few studies evaluating the use of intracranial VW-MRI specifically in the ESUS/CS population (Table 3). A 7 Tesla (T) VW-MRI study in 2020 by Fakih et al., evaluated 34 patients admitted to the stroke service with acute stroke of cryptogenic origin and unspecific arterial changes on CTA, MRA or DSA that did not meet a diagnosis of a specific vasculopathy. 7T VW-MRI led to the determination of a new stroke etiology in 28 of 34 patients, among which intracranial atherosclerosis was adjudicated as the stroke etiology in 25 of 28 patients. 7T VW-MRI identified culprit plaques as having significantly higher plaque-to-pituitary stalk contrast enhancement ratio, showed concentric morphology compared to non-culprit plaques, showed higher mean signal intensity, and higher contrast ratios compared to non-culprit plaques (*p* ≤ 0.001) (62). This study suggests 7T VW-MRI may be used to achieve a more accurate diagnosis of underlying intracranial atherosclerosis as a stroke etiology in a subset of stroke patients meeting criteria for cryptogenic stroke (Figure 7).

**Table 3:** Select studies using VW-MRI in an ESUS/CS population for evaluation of intracranial atherosclerosis.

**Study**	**Imaging technique**	**Vessel**	**Key imaging characteristics**	**Select results**
Fakih et al. (62)	7T. 3D TOF MRA, 3D T1W fast-spin-echo (CUBE), T2W CUBE, 3D susceptibility-weighted angiogram, post-contrast 3D TIW CUBE	Supraclinoid ICA, MCA, ACA, VA, Basilar, PCA	Contrast enhancement; plaque-to-pituitary stalk contrast enhancement ratio (CR), degree of stenosis, morphology	Culprit plaques (*n* = 36) had higher CR and had concentric morphology than non-culprit plaques (*p* ≤ 0.001). CR ≥53 (*p* = 0.008), stenosis ≥50% (*p* < 0.001), and concentric morphology (*p* = 0.030) as independent predictors of culprit plaques.
Tao et al. (8)	3T. 3D TOF MRA, 3D T1W CUBE, 2D T2W	ICA, MCA, ACA, VA, Basilar artery, PCA	Remodeling index (RI), plaque burden, plaque surface discontinuity, thick fibrous cap, IPH	Higher prevalence of intracranial plaque ipsilateral than contralateral side of ischemic stroke [63.8% vs. 42.8%; odds ratio (OR): 5.25; 95% CI: 2.83–9.73]. RI independently associated with ESUS; model 1 (OR: 2.329; 95% CI: 1.686 −3.217; *p* < 0.001) and model 2 (OR: 2.295; 95% CI: 1.661–3.172; *p* < 0.001).

*VW-MRI, vessel wall MRI; T1W GRE, T1 weighted gradient-echo; T, Tesla; TOF MRA, Time-of-flight MR angiography; PD, proton density; T2W, T2 weighted; ICA, internal carotid artery; MCA, middle cerebral artery; ACA, anterior cerebral artery; VA, vertebral artery; PCA, posterior cerebral artery; IPH, intraplaque hemorrhage; CR, plaque-to-pituitary stalk contrast enhancement ratio; RI, remodeling index; 95% CI, 95% confidence interval*.

A 3T VW-MRI study by Tao et al. (8) evaluated the morphology and composition of intracranial plaque in the ESUS population compared to patients with small-vessel disease (SVD). The authors hypothesized a higher prevalence of non-stenotic plaque with positive wall remodeling, which is more prone to vulnerability and rupture, on the ipsilateral side of stroke in ESUS patients. Among 243 patients with ESUS, the prevalence of intracranial plaque ipsilateral to ESUS was significantly higher compared to the contralateral side (63.8% vs. 42.8%; odds ratio 5.25, 95% CI 2.83–9.73). In comparison, among 160 patients with SVD, there was no significant difference in the prevalence of intracranial plaques between the ipsilateral/contralateral sides. Vulnerable plaque features significantly more prevalent on the ipsilateral side of ESUS included positive remodeling and discontinuity of plaque surface (DPS; e.g., surface irregularity suggesting an ulcer, fibrous cap rupture, or formation of overlying mural thrombus), a plaque finding not evident in patients with SVD. The authors concluded that high-risk non-stenotic intracranial plaque may represent a significant but currently underestimated embolic source of ESUS.

### Future directions

The use of intracranial VW-MRI to evaluate intracranial atherosclerosis as the culprit source of ischemic stroke is in its early stages. Challenges toward clinical adoption may be related to lengthy acquisition times, variability in VW-MRI protocols and pulse sequence designs, and lack of histologic validation studies (90). Given challenges in obtaining histologic specimens, serial imaging to evaluate the progression of vessel wall changes may be useful to better understand the role of MRI in plaque characterization.

**Figure 8:**
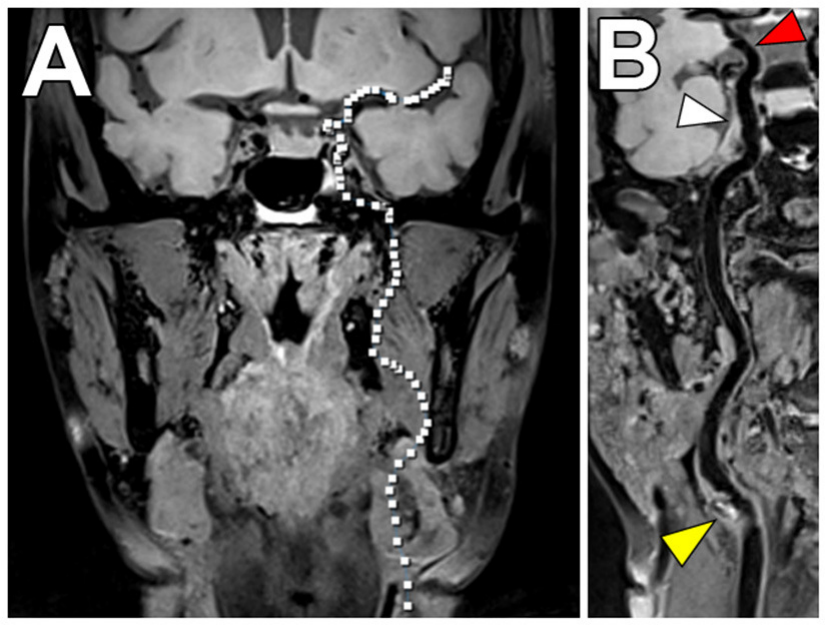
Joint VW-MRI of the extracranial and intracranial arteries. **(A)** Coronal precontrast VW-MRI image shows the coverage of the joint intracranial and extracranial VW-MR image. **(B)** A curved reformatted image of the left cervico-cranial carotid artery shows intrinsic T1 hyperintense signal at the carotid bulb (yellow arrowhead), which was favored to be the culprit source of plaque in this patient with ischemic strokes. The intracranial internal carotid artery at the siphon (white arrowhead) and M1 middle cerebral artery segment (red arrowhead) showed no significant wall thickening to suggest intracranial atherosclerosis.

Many patients with vascular risk factors will have systemic atherosclerosis and have disease involving both the intracranial and cervical carotid arteries (91). To address this, investigators have proposed VW-MRI pulse sequences that permit joint intracranial and extracranial artery evaluation (Figure 8). Technical considerations for these pulse sequences include attention to the (1) needed spatial resolution to evaluate the smaller intracranial artery walls compared to the extracranial carotid artery walls, (2) need for adequate cerebrospinal fluid suppression around the circle of Willis compared to fat suppression around the carotid arteries, and (3) head/neck coils that allow adequate coverage. These innovative joint intracranial-extracranial VW-MRI pulse sequences provide promise in identifying the culprit source of stroke in vulnerable patients with systemic atherosclerosis and multiple potential sources of stroke (92–95).

**Figure 9:**
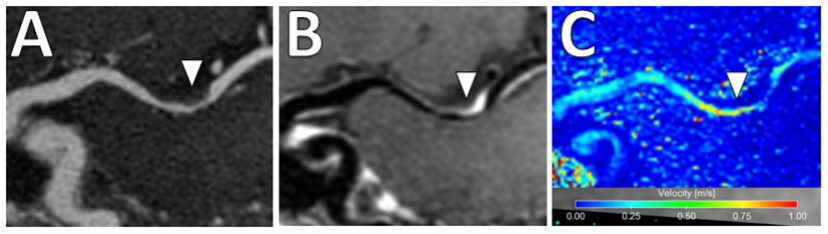
4D Flow MRI of intracranial atherosclerosis. **(A)** TOF MRA image demonstrates a focal area of middle cerebral artery narrowing (arrowhead) with **(B)** post-contrast T1-weighted VW-MRI showing a corresponding eccentric, enhancing atherosclerotic plaque (arrowhead). **(C)** On 4D Flow MRI, increased velocities are seen in the area of greatest narrowing (arrowhead).

In addition, there is increased interest in characterizing velocity using 4D Flow MRI. This is an advanced phase-contrast MRI technique which allows non-invasive quantifications of blood flow to characterize the hemodynamic impact of intracranial atherosclerosis and identify hemodynamic biomarkers (96) (Figure 9). While studies to date share limitations including long scan duration, low spatial/temporal resolutions and associated tradeoffs (97), this is a promising emerging technique.

## Aortic arch

### Background

Observational studies have identified several morphological features of aortic arch atherosclerosis associated with a high risk of stroke recurrence. Plaque thickness ≥4 mm was observed to be an independent predictor of recurrent stroke and new arterial vascular events (98). Plaque ulcerations and the presence of mobile thrombi were also shown to be high-risk features of aortic arch atherosclerosis (99, 100). Given these results, the ASCOD Phenotyping of Ischemic Stroke, which is an etiological classification system for ischemic stroke proposed in 2013, classifies aortic arch atherosclerosis with mobile thrombus and plaque ≥4 mm without a mobile thrombus as possible causes of stroke (15).

Aortic arch atherosclerosis is an often unrecognized stroke mechanism and a potentially important contributor to ESUS (1). In an exploratory analysis of the NAVIGATE ESUS trial, 29% of ESUS patients who underwent evaluation of the aortic arch by transesophageal echocardiography (TEE) had aortic arch atherosclerosis of any severity, 21% had non-complex aortic arch atherosclerosis, and 8% had complex aortic arch atherosclerosis (9). “Complex plaques” included plaques with ulcerations or ≥4 mm in wall thickness or had a mobile thrombus (9). A prospective case-control study by the French Aortic Plaque in Stroke (FAPS) group used TEE to quantify the risk of ischemic stroke with arch atherosclerosis. The authors showed an adjusted odds ratio of 9.1 (3.3–25.2) among 250 cases and 250 controls of a risk of cerebral infarction with arch/proximal plaque thickness ≥4 mm (101).

The dominant imaging modality used to evaluate the aorta in most centers currently is TEE (102). TEE has high sensitivity and specificity for aortic arch atherosclerosis and has sufficient image quality to allow measurement of plaque thickness as well as detection of ulceration and mobile thrombus (103). Limitations of TEE include its invasive nature and operator-dependence. Specifically in regards to aortic plaque imaging, the aortic wall cannot be visualized in its entirety on TEE due to near-field signal losses (104) and limited anatomic evaluations of the ascending aorta due to tracheal and bronchial artifacts (105). Given these limitations, investigations using cross-sectional imaging modalities, such as CT and MRI, have started to emerge (102). Recent advances in 3D-multi-contrast MRI for the detection of aortic atherosclerosis has enabled characterizations of plaque compositions and identification of vulnerable plaques (106, 107).

### MRI of vulnerable aortic arch atherosclerosis features

#### Plaque thickness

Both 1.5 and 3T MR studies imaging the aortic arch have been compared to TEE with respect to plaque thickness and are summarized in Table 4. Comparing 1.5T MR and TEE, Kutz et al. (108), reported the mean atheroma size in the aortic arch was underestimated on MR compared to TEE (3.4 ± 3.1 mm by TEE, 1.4 ± 3.0 mm by MRA, *P* = 0.01). However, Fayad et al. (109), reported strong correlation and agreement for both 1.5T MR and TEE when measuring maximum plaque thickness and no significant difference in the measurements values (4.62 ± 0.31 mm by TEE, 4.56 ± 0.21 mm by MRI, *r* = 0.88).

**Table 4:** Comparison of transesophgeal echocardiogram vs. MR imaging of the aortic arch/proximal aorta evaluating plaque thickness.

**Study**	**Imaging technique**	**Aortic segment**	**Key imaging characteristics**	**Select results**
Kutz et al. (108)	1.5T. 3D MRA with T1W vs. TEE; patients imaged with both modalities within 1 month	Asc, Arch, Desc	Plaque size/thickness	*N* = 30 patients; Plaques measuring ≥5 mm, 22 (92%) were seen on TEE and only 13 (54%) on MRA, *p* = 0.003
Fayad et al. (109)	1.5T. T1W, PDW, T2W vs. TEE; patients imaged with both modalities within 39 ± 13 days	Desc	Max plaque thickness, plaque extent, plaque composition	*N* = 10 patients, 25 plaques; Strong correlation or agreement between modalities for max plaque thickness (*r* = 0.88, *n* = 25; 4.56 ± 0.21 mm by MR and 4.62 ± 0.31 mm by TEE), plaque extent [χ^2^ = 61.77, *p* < 0.0001; 80% overall agreement] and plaque composition [χ^2^ = 43.5, *p* < 0.0001; 80% overall agreement]
Harloff et al. (110)	3T. ECG-synchronized pre- and post-contrast 3D T1W FS, 2D T2W, time resolved (CINE) imaging vs. TEE; patients imaged with both modalities within 5–6 days (median)	Asc, Arch, Desc	Max wall thickness of high risk plaques (≥4 mm or superimposed thrombi)	*N* = 74 patients; No significant difference between MRI and TEE measurements of plaque thickness; Strong agreement in detection of high risk plaques (0.90 > κ > 0.50); CINE imaging allowed for detection of mobile thrombus
Harloff et al. (111)	3T. 3D contrast-enhanced MRA, ECG-gated 3D T1W RF-spoiled FS bright-blood GRE, 2D T2W TSE, 3D CINE T1W vs. TEE; patients imaged with both modalities within 3 days (median)	Asc, Arch, Desc	Complex plaques (≥4 mm thick, ulcerated, or with mobile thrombus)	*N* = 99 patients. MRI detected more complex plaques than TEE (Asc, 13 vs. 7; Arch, 37 vs. 11; Desc, 101 vs. 70). Image quality was higher for MRI in Asc and Arch and higher for TEE in Desc

*Asc, ascending, Desc, descending; FS, fat suppressed; MRA, magnetic resonance angiography; TEE, transesophageal echocardiogram*.

Using 3T MR showed more promising results between TEE and MRI and advantages with MRI. Harloff et al. (110), compared the maximum aortic wall thickness measured by TEE against T1W sequence and reported the wall of the aortic arch was not reliably assessed by TEE because of limited visualization in 45 of 74 patients. Among those who were able to be measured, the maximum wall thickness of the aortic arch did not have a statistically significant difference between the modalities (3.36 ± 1.46 mm by TEE, 2.83 ± 1.37 by MRI, *p* = NS). A subsequent study by Harloff et al. (111), used the same MRI acquisition protocol and showed that MRI detected more complex plaques than TEE at the aortic arch (MRI 37 vs. TEE 11, *p* = 0.003). Notably, the authors reported MRI may potentially overestimate the wall thickness (mean plaque thickness in MRI 5.2 ± 1.1 95% CI 4.1–8.4 vs. TEE 4.7 ± 0.8 95% CI 4.0–6.5).

**Figure 10:**
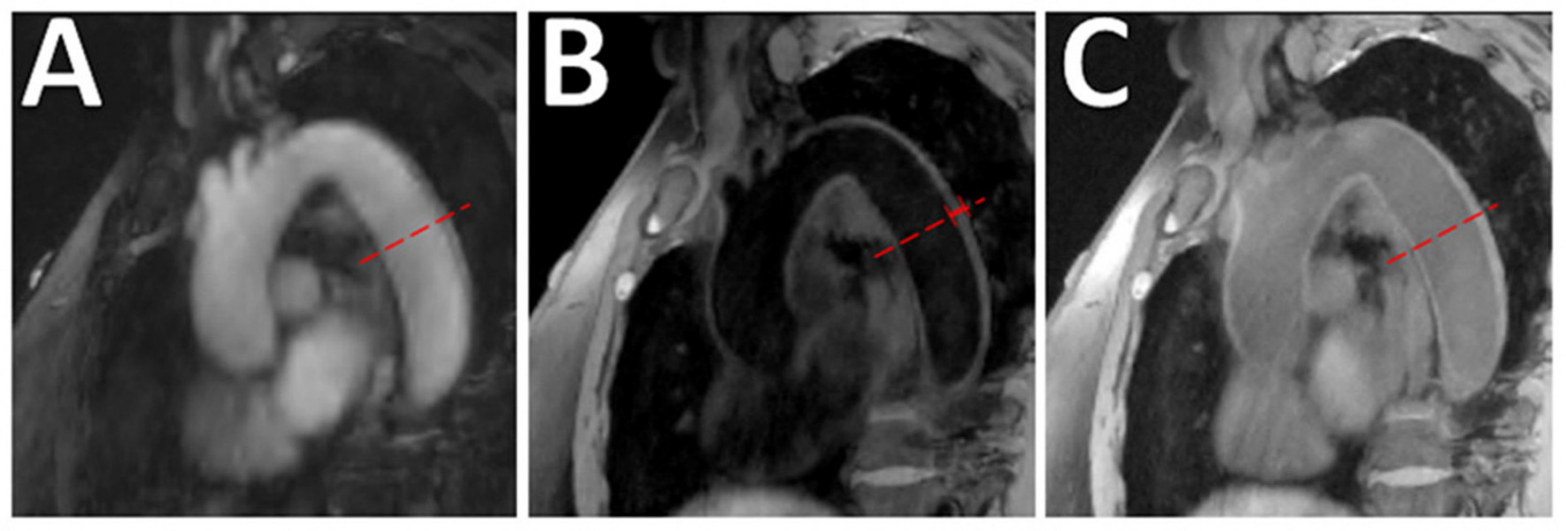
Aortic VW-MRI using the MR MultiTasking based 3D Multidimensional Assessment of Cardiovascular System (MT-MACS) Technique. **(A)** In a 71-year-old patient with aortic atherosclerosis, bright-blood lumenography, **(B)** dark-blood (vessel wall imaging), and **(C)** gray-blood (optimized to detect calcium/calcified nodules) images were acquired. In the thoracic aorta (dashed line), increased aortic wall thickness (4.491 mm) was measured and most conspicuous on the dark-blood **(B)** and gray-blood **(C)** images. [These images are re-printed with permission from Zhaoyang Fan, PhD from Magnetic Resonance in Medicine (112).]

The long acquisition times for performing multiple acquisitions and the need for ECG-gating and respiratory navigations have resulted in a slow adoption of aortic VW-MRI techniques. Newer innovations in the aortic VW-MRI space show the ability to simultaneously image both the lumen and vessel wall integrity (112, 113). One technique that circumvents the need for these is the MR MultiTasking (MT) based 3D Multi-dimensional Assessment of Cardiovascluar System (MCAS) technique which allows for motion-resolved, isotropic high-spatial resolution, multi-dimensional (multiple contrast weights and cine images) imaging of the thoracic aorta in 6 min (112) (Figure 10). Due to its non-invasive nature, MRI is becoming increasingly used to investigate surveillance imaging for atherosclerosis treatment (114, 115).

#### Plaque ulceration

An autopsy study showed a higher prevalence of ulcerated aortic plaques with depth and width ≥2 mm in cryptogenic stroke patients (99) and a subsequent study observed that ulcerated plaques were detected by TEE more frequently in patients with cryptogenic stroke than in patients with known-cause strokes or age-matched controls without stroke (116). When directly compared to TEE, 3D MRI has demonstrated mixed results regarding its ability in detecting ulceration. One study using 3D MRI observed limited ability of MRI in detecting ulceration compared to TEE (MRI detected 89% of proximal aorta ulcerations and 64% of distal aorta ulceration) (117). Meanwhile, another 3D MRI study reported MRI detected more plaques with ulcerations compared to TEE (111). These differences may be due to differences in MRI techniques, such as magnet strength (1.5T vs. 3 T MRI) as well as small sample sizes (22 vs. 74) and thus additional explorations are warranted.

#### Mobile thrombus

Recent advancements in cardiac MRI techniques such as ECG-gated CINE imaging, which is a type of MRI sequence to capture motion, has allowed evaluation of mobile thrombus by MRI (110, 111). In these studies, structures within the blood stream that were mobile on CINE imaging were defined as mobile thrombus.

#### Plaque compositions

Similar to investigative aims for VW-MRI of plaque in the cervical carotid and intracranial arteries, aortic VW-MRI has been used to investigate plaque compositions. Yamaguchi et al. (118), performed a retrospective study of 135 patients with ischemic stroke/TIA and imaged on a 3T MRI using a T1W MPRAGE fat suppressed pulse sequence of the aortic arch. Detection of high signal intensity (>200% of sternocleidomastoid muscle signal intensity) on T1W MPRAGE was considered a vulnerable feature and compared to aortic complicated lesions (ACLs) detected by TEE. The results showed that high intensities on MPRAGE were independently associated with ACLs (OR 5.72, 95% CI 2.38–13.70).

Morihara et al. (119), applied a technique called liver-acquisition-with-volume-acceleration-flexible (LAVA-Flex) to assess the relationship between high-intensity plaque lesions in cervical carotid and aortic plaques and plaque thickness on TEE. High-intensity carotid plaque lesions detected on LAVA-Flex were histologically validated on carotid endarterectomy specimens to be large lipid cores and hemorrhage. Hyperintense lesions detected in the thoracic aorta on LAVA-Flex were observed in 24 (51.1%) of 47 CS patients. Twenty-one (87.5%) of these hyperintense aortic lesions also showed a ≥4 mm plaque on TEE, which was considered the gold standard. LAVA-Flex showed a sensitivity of 95.5% and a specificity of 88.0% in patients with large aortic plaques (≥4 mm thickness).

### Literature on aortic arch atherosclerosis VW-MRI in ESUS/CS patients

Literature on the use of MRI to study the prevalence of complex aortic arch atherosclerosis specifically in the ESUS/CS populations is scarce and summarized in Table 5. Harloff et al. (110), reported up to one-third of patients initially to have CS were re-categorized with a high risk aortic plaque after undergoing aortic VW-MRI. In a recent study comparing 40 CS patients to 60 controls, plaques <4 mm thickness were found in similar numbers in CS patients and controls [23 (57.5%) vs. 33 (55.0%); *p* = 0.81] but plaques ≥4 mm were more frequent in CS patients [22 (55.0%) vs. 10 (16.7%); *p* < 0.001] (107).

**Table 5:** Select studies using VW-MRI in an ESUS/CS population for evaluation of aortic arch atherosclerosis.

**Study**	**Imaging technique**	**Vessel**	**Key imaging characteristics**	**Select results**
Harloff et al. (110)	3T. ECG-synchronized pre- and post-contrast 3D T1W FS, 2D T2W, time resolved (CINE) imaging	Asc, Arch, Desc	Max Wall Thickness of high risk plaques (≥4 mm or superimposed thrombi)	MRI identified high risk pathologies in 8 of 26 (30.8%) CS patients after standard diagnostic work-up including TEE
Harloff et al. (111)	3T. 3D contrast-enhanced MRA, ECG-gated 3D T1W RF-spoiled FS bright blood GRE, 2D T2W TSE, 3D CINE T1W	Asc, Arch, Desc	Complex plaques (≥4 mm thick, ulcerated, or with mobile thrombus)	MRI showed additional complex plaques in 19 of 58 (32.8%) CS patients after diagnostic work-up including TEE
Wehrum et al. (107)	3T. 3D T1W bright-blood, 3D T2W black-blood, 3D PD black-blood. 4D flow used to visualize 3D blood flow within the thoracic aorta	Asc, Arch, Desc	Plaque thickness, surface irregularities, thrombus, IPH, calcification, fibrous tissue	Plaques ≥4 mm more frequent in CS patients than controls [22 (55.0%) vs. 10 (16.7%); *p* < 0.001]. Of those with plaques ≥4 mm, AHA-LT VI plaques higher in stroke patients (17.5% vs. 3.3%, *p* < 0.001). No significant difference between groups for plaques <4 mm thickness [23 (57.5%) vs. 33 (55.0%); *p* = 0.81]
Jarvis et al. (120)	1.5T. 4D flow MRI and 3D T1W black-blood TSE	Mid-Asc, Arch, proximal-Desc, distal-Desc	Aortic wall thickness (3D T1W black-blood), pulse wave velocity (PWV, measure of arterial stiffness) and voxel-wise mapping of flow reversal fraction (FRF)	Aortic PWV and FRF higher in CS patients (8.9 ± 1.7 m/s, 18.4 ± 7.7%) than younger controls (5.3 ± 0.8 m/s, *p* < 0.0167; 8.5 ± 2.9%, *p* < 0.0167), but not age-matched controls (8.2 ± 1.6 m/s, *p* = 0.22; 15.6 ± 5.8%, *p* = 0.22). Maximum aortic wall thickness higher in CS patients (3.1 ± 0.7 mm) than younger controls (2.2 ± 0.2 mm, *p* < 0.0167) and age-matched controls (2.7 ± 0.5 mm) (*p* < 0.0167)

*VW-MRI, vessel wall MRI; T1W, T1 weighted; T, Tesla; RF, radiofrequency; FS, fat suppressed; PD, proton density; T2W, T2 weighted; CE MRA, contrast-enhanced magnetic resonance angiography; IPH, intraplaque hemorrhage; AHA-LT VI, American Heart Association lesion type VI; PWV, pulse wave velocity; FRF, flow reversal fraction; 95% CI, 95% confidence interval; Asc, Ascending aorta; Desc, Descending aorta; TSE, turbo spin echo; CS, cryptogenic stroke*.

### Future directions

Interest in the use of MRI to evaluate aortic arch atherosclerosis in acute ischemic stroke patients is re-emerging. VW-MRI techniques used for cervical carotid and intracranial arteries may have reignited an interest in characterizing vulnerable aortic plaque features as a potential source for ESUS. Features of aortic arch atherosclerosis that have traditionally been used to define “complex plaques” for risk stratification in acute ischemic stroke patients, such as plaque thickness, ulcerations, and mobile thrombus, are mostly derived from studies using TEE as the main diagnostic imaging modality. However, MR evaluation of the aorta in CS/ESUS patients may be complementary to TEE (110, 111) by offering additional specificity in detecting aortic sources of embolism in patients who would otherwise be labeled as having CS (121, 122).

Advanced MR imaging techniques such as multicontrast VW-MRI, 4D Flow MRI, and CINE imaging are increasingly being explored. 4D Flow MRI allows analysis of hemodynamic flow parameters and can be used to investigate not only aortic wall thickness and stiffness but also flow reversal in the proximal descending aorta in patients with ESUS/CS given retrograde embolic mechanisms during diastole may explain a portion of ESUS/CS (120, 123–125). As technical feasibility is established using these advanced imaging techniques, more studies examining ESUS/CS populations are anticipated to emerge in the future. These advanced imaging techniques may have future applications in the evaluation of aortic arch atherosclerosis in the ESUS population and are promising avenues of research for this vulnerable population.

## Conclusion

Despite advancements in stroke diagnostics, a considerable proportion of acute ischemic stroke patients remain without a clear determined mechanism. There has been increased attention to decoding the contributory role of non-stenotic atherosclerosis in the carotid arteries, intracranial arteries, and the aortic arch, in ESUS patients. Non-invasive methods to detect such atherosclerotic plaques and evaluate their morphologies for risk stratification is desirable, and the use of VW-MRI is increasingly becoming investigated.

The preponderance of existing literature on the use of VW-MRI for assessment of vulnerable plaque focuses on the cervical carotid arteries, with increasing translation to the intracranial arteries and the aortic arch. High-risk features of carotid artery atherosclerosis such as IPH, LRNC and fibrous cap status are concepts derived from earlier work in the coronary arteries, and it has been demonstrated that VW-MRI is capable of detecting these features. For intracranial atherosclerosis, additional VW-MRI imaging features have been proposed such as contrast enhancement, vessel wall thickening and vessel wall remodeling; further evaluation in ESUS patients is needed to firmly establish histologic correlation and potential for risk stratification. For aortic arch atherosclerosis, the conventional definitions of “high-risk” features are those derived from earlier non-MRI studies. VW-MRI allows further characterization of aortic arch plaque compositions similar to that seen in carotid studies. Given the rapid advancement of VW-MRI techniques, further studies may demonstrate novel imaging features in aortic arch atherosclerosis.

The use of VW-MRI to detect and characterize carotid, intracranial, and aortic arch atherosclerosis in ESUS patients is an exciting and rapidly evolving field. Additional efforts are warranted to elucidate the contributory role of these atherosclerotic plaques in ESUS.

## Author contributions

YS: writing and original draft. VL, LE, EO, and ZF: contributions of images and manuscript review and editing. GK, JX, GW, and BC: manuscript review and editing. JS: conceptualization, contributions of images, and manuscript review and editing. All authors contributed to the article and approved the submitted version.

## Funding

The work is supported by the NINDS (L30 NS118632) (JS), Vice Provost for Research University Research Foundation (JS), NHLB (R01 HL147355) (ZF), the Clinical and Translational Science Award program/NIH National Center for Advancing Translational Sciences UL1TR002373 and KL2TR002374 (LE), and Wisconsin Alzheimer's Disease Research Center grant P30-AG062715 (LE).

## Conflict of interest

The authors declare that the research was conducted in the absence of any commercial or financial relationships that could be construed as a potential conflict of interest.

## Publisher's note

All claims expressed in this article are solely those of the authors and do not necessarily represent those of their affiliated organizations, or those of the publisher, the editors and the reviewers. Any product that may be evaluated in this article, or claim that may be made by its manufacturer, is not guaranteed or endorsed by the publisher.
